# From Allergy to Angina: A Unique Presentation of Kounis Syndrome

**DOI:** 10.1002/ccd.31522

**Published:** 2025-03-27

**Authors:** Momen Ali, Ayman Helal, Mohammad El‐Din, Ibrahim Antoun

**Affiliations:** ^1^ Department of Cardiology Kettering General Hospital Kettering UK; ^2^ Department of Cardiovascular Sciences University of Leicester Leicester UK

**Keywords:** angioedema, acute coronary syndrome, allergic reaction, coronary angiogram, kounis syndrome

## Abstract

Kounis syndrome (KS) is a rare condition characterized by acute coronary syndrome (ACS) triggered by an allergic reaction. This report presents a case of high‐risk ACS associated with a food allergy. A 53‐year‐old male with no prior medical history presented to the emergency department with itching, facial swelling, chest tightness, shortness of breath, and presyncope after consuming peanut butter and grapefruit juice. His past medical history included an allergy to codeine/paracetamol, causing angioedema. Initial vitals were stable, and examination revealed minimal lip swelling, a pruritic rash, and clear auscultation. A baseline electrocardiogram (ECG) demonstrated subtle ST‐segment depression with T‐wave inversion in inferior leads, which progressed to significant ST depression and deep T‐wave inversion. Serial troponin levels showed a significant rise (20.2 to 39.2 ng/L). Coronary angiography revealed no significant coronary artery disease. Cardiac magnetic resonance (CMR) excluded myocardial infarction or fibrosis. The patient was diagnosed with KS based on clinical presentation, dynamic ECG changes, and elevated troponins in the absence of obstructive coronary artery disease. Management included antihistamines, steroids, nitroglycerin, and standard acute coronary syndrome treatment. He was discharged on oral antihistamines after a brief coronary care unit observation.

## 1 Introduction

Kounis syndrome (KS) is a rare yet significant clinical condition that illustrates the complex interplay between allergic reactions and acute coronary syndrome (ACS). First described in 1991 by Kounis and Zavras, the syndrome represents an intriguing overlap of two seemingly unrelated pathological processes: an allergic inflammatory response and coronary vasospasm or plaque rupture [1]. While its prevalence is estimated at 1.1% among hospitalized patients with allergic reactions, the incidence may be underestimated due to underrecognition and diagnostic challenges [2].

Various allergens trigger the syndrome, including medications, insect stings, and certain foods. It is mediated by the release of inflammatory substances such as histamine, leukotrienes, and cytokines, which cause coronary artery vasospasm and, in some cases, plaque destablization. Clinical presentations range from mild chest discomfort to life‐threatening ACS, with electrocardiogram (ECG) changes varying from ST‐segment elevation to depression and T‐wave inversion.

This case report highlights a unique presentation of high‐risk ACS in the context of a severe allergic reaction triggered by food ingestion. By detailing the diagnostic process, management strategies, and clinical course, this report emphasizes the importance of considering KS in patients with simultaneous allergic and cardiac symptoms. Early recognition and tailored treatment are crucial in preventing adverse outcomes, particularly given the challenges in distinguishing KS from other causes of ACS. This case underscores the need for heightened awareness of this underdiagnosed entity among clinicians.

## 2 Case Presentation

**Figure 1:**
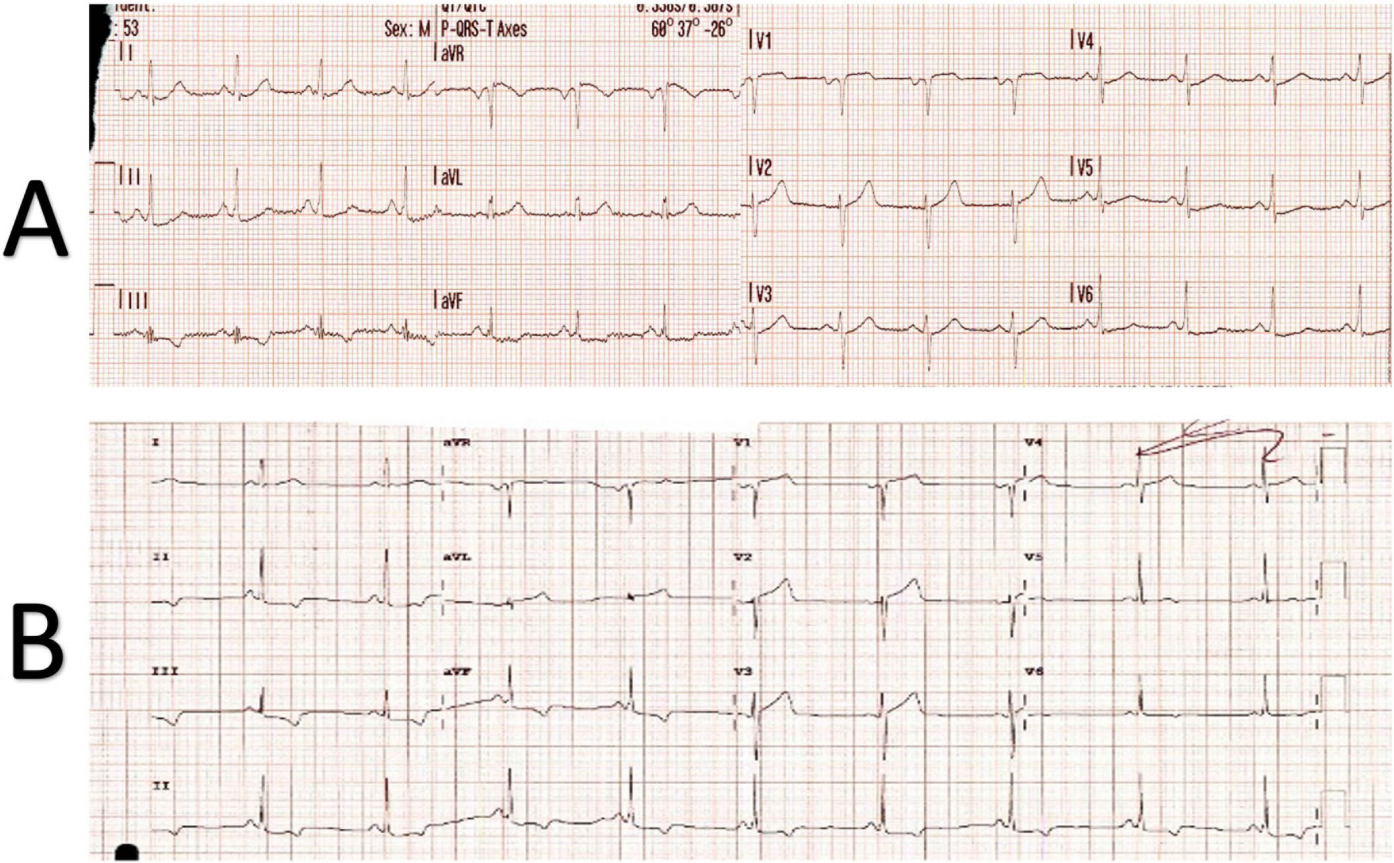
(A) the presenting 12 leads electrocardiogram showing sinus rhythm with subtle 0.5 mm ST segments depression and T wave inversion in inferior leads. (B) The second electrocardiogram shows dynamic ECG changes with significant ST depression and deep T inversion in the inferior leads.

**Figure 2:**
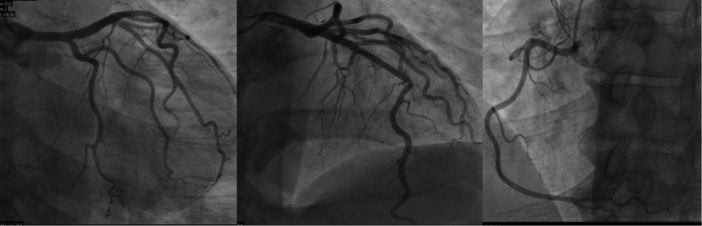
Invasive coronary angiogram showing no clear coronary artery disease.

A 53‐year‐old male with no previous medical history presented to the emergency department with a chief complaint of generalized itching and face swelling shortly after ingesting peanut butter and grapefruit juice. These symptoms were followed by central chest tightness, shortness of breath and presyncope. The patient had a history of allergy to codeine/paracetamol combination tablets in the form of angioedema. The ambulance crew administered IV antihistamines. On admission, blood pressure was 108/66 mmHg, heart rate was 91 beats per minute, oxygen saturation was 94% on room air and 21 respirations per minute. On examination, the patient was alert and oriented, with minimal lip swelling (interval improvement as per the patient) and an itchy skin rash (hives). The airway was patent. A cardiac examination revealed a good bilateral radial pulse with no heart murmurs and clear lung auscultation. Abdominal examination was unremarkable, and there were no signs of systemic anaphylaxis, such as stridor or hypotension. Baseline ECG (Figure 1A) showed normal sinus rhythm with subtle 0.5 mm ST‐segment depression and T wave inversion in inferior leads. Subsequent ECG 2 h afterwards showed dynamic ECG changes with significant ST depression and deep T inversion in the inferior lead (Figure 1B). Highly sensitive Troponin was 20.2 ng/L (reference < 12 ng/L), rising to 39.2 ng/L 2 h afterwards. Chest X‐ray was unremarkable. An echocardiogram showed a structurally normal heart with preserved biventricular systolic function without regional wall motion abnormalities. Serum tryptase was elevated at 16.8 µg/L (reference < 11.4 µg/L), consistent with mast cell activation and supporting an allergic aetiology. The decision was made to take the patient urgently to the cardiac catheterization laboratory because of the chest pain, the dynamic ECG changes and the elevated troponin. A coronary angiogram revealed no obstructive coronary artery disease (Figure 2). Type 1‐KS was diagnosed based on the following criteria:

1. Clinical Presentation: Acute chest pain and shortness of breath following peanut butter ingestion, with associated allergic symptoms (itchy rash and face swelling).
2. Cardiac Involvement: ST‐segment depression and elevated troponin levels.
3. Absence of significant coronary artery disease**:** Normal coronary angiography.
4. Elevated serum tryptase.

The patient was initially managed with antihistaminic and steroids IV with standard ACS initial management (dual antiplatelet, anticoagulation in the form of low molecular weight heparin and Nitroglycerin). The patient was observed in the coronary care unit (CCU) and subsequently discharged 48 h later with an oral antihistamine.

**Figure 3:**
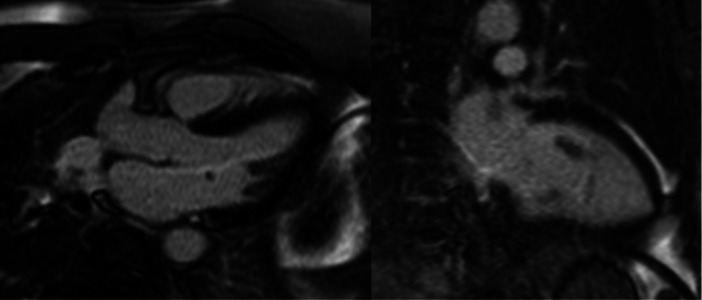
The heart's Magnetic resonance after the event demonstrates the lack of late gadolinium enhancement.

The cardiac magnetic resonance imaging (MRI) showed no late gadolinium enhancement with normal T1 and T2 Mapping values, excluding myocardial infarction, oedema and fibrosis (Figure 3). The patient remains asymptomatic after avoiding peanuts. The patient subsequently underwent allergy testing by the allergy specialist, which confirmed a strong positive IgE‐mediated reaction to peanuts, while other tested allergens, including grapefruit, were negative.

## 3 Discussion

In severe allergic reactions, many inflammatory mediators, especially histamine, are released in the bloodstream, causing peripheral vasodilatation; however, histamine can act on the coronary histamine receptors, leading to coronary vasospasm [3]. Coronary vasospasm is one of the manifestations of KS. ECG changes in KS can vary between ST elevation, depression and T wave inversion. ST elevation is considered the most common change encountered [4]. Patients with KS present with angina, dyspnoea or even sudden death [4]. Up to 31% of patients presenting with KS have documented allergies [5]. KS is classified into three types: Type 1, occurring in patients with normal coronary arteries due to coronary vasospasm; Type 2, seen in patients with pre‐existing coronary artery disease where an allergic reaction triggers plaque rupture and thrombosis [6]. Lastly, type 3 KS includes instent thrombosis [7]. Our case represents Type 1 KS, as no obstructive coronary artery disease was identified on angiography.

This case was further supported by elevated tryptase levels and allergy testing confirming a peanut allergy, strengthening the diagnosis of food‐induced KS. The temporal proximity of peanut ingestion and symptoms onset is suggestive of an allergic trigger.

Diagnosing KS can be challenging. The Tryptase level can be checked 2 h after the onset of symptoms and repeated later for the uptrend. CMR and thallium‐201 single‐photon emission computer tomography can aid in diagnosis as well [6].

It is important to consider KS among the other differential diagnoses for chest pain, especially in patients presenting with ACS in the context of allergic reactions. Vasodilators such as Nitroglycerin and calcium channel blockers can be used to treat coronary vasospasm in haemodynamically stable patients [7]. Antihistamines and corticosteroids can be used to counter the allergic reaction [7].

Our case report adds to the literature describing Type 1 KS induced by food allergy. Given the overlap between allergic reactions and cardiac events, clinicians should consider KS as part of the differential diagnosis when faced with similar presentations.

## 4 Conclusion

This case report highlights the complexity of diagnosing and managing KS, a rare yet clinically significant overlap between allergic reactions and ACS. The patient presented with severe allergic symptoms and high‐risk ACS features, underscoring the importance of early recognition and tailored management of this underdiagnosed condition. The clinical presentation, dynamic ECG changes, elevated troponin, and absence of significant coronary artery disease on angiography supported the diagnosis of KS. Management included a combination of antihistamines, corticosteroids, and traditional ACS treatment.

This case emphasizes the critical need for clinicians to consider KS in patients with concurrent allergic and cardiac symptoms, particularly given its diagnostic challenges. Early identification and appropriate intervention can prevent adverse outcomes and improve patient care. Increased awareness of KS and its clinical spectrum is essential to optimize diagnosis and treatment strategies in clinical practice.

## Consent

The authors confirm that written consent was obtained before submission of the case report.

## Conflicts of Interest

The authors declare no conflicts of interest.
